# Isolation and Identification of Chemical Constituents from Zhideke Granules by Ultra-Performance Liquid Chromatography Coupled with Mass Spectrometry

**DOI:** 10.1155/2020/8889607

**Published:** 2020-12-27

**Authors:** Guangqiang Huang, Jie Liang, Xiaosi Chen, Jing Lin, Jinyu Wei, Dongfang Huang, Yushan Zhou, Zhengyi Sun, Lichun Zhao

**Affiliations:** ^1^College of Pharmacy, Guangxi University of Chinese Medicine, Nanning 530200, China; ^2^Guangxi Key Laboratory of Zhuang and Yao Ethnic Medicine, Nanning 530200, China; ^3^Guangxi Zhuang Yao Medicine Center of Engineering and Technology, Nanning 530200, China; ^4^Ruikang Hospital Affiliated to Guangxi University of Chinese Medicine, Nanning 530011, China

## Abstract

Chemical constituents from Zhideke granules were rapidly isolated and identified by ultra-performance liquid chromatography (UPLC) coupled with hybrid quadrupole-orbitrap mass spectrometry (MS) in positive and negative ion modes using both full scan and two-stage threshold-triggered mass modes. The secondary fragment ion information of the target compound was selected and compared with the compound reported in databases and related literatures to further confirm the possible compounds. A total of 47 chemical constituents were identified from the ethyl acetate extract of Zhideke granules, including 21 flavonoids and glycosides, 9 organic acids, 4 volatile components, 3 nitrogen-containing compounds, and 10 other compounds according to the fragmentation patterns, relevant literature, and MS data. The result provides a new method for the analysis of chemical constituents of Zhideke granules which laid the foundation for quality control and the study of pharmacodynamic materials of Zhideke granules.

## 1. Introduction

Zhideke granules are an in-hospital preparation from Ruikang Hospital affiliated to the Guangxi University of Chinese Medicine. It contains 10 kinds of traditional Chinese medicines including *Scutellaria baicalensis* Georgi., *Belamcanda chinensis* (L.) Redouté, *Mentha haplocalyx* Briq., *Eriobotrya japonica* (Thunb.), *Platycodon grandiflorus* (Jacq.) A. DC., *Bupleurum chinense*, *Nepeta cataria* L., *Cynanchum glaucescens* (Decne.) Hand-Mazz., *Nervilia fordii* (Hance) Schltr., and *Sauropus spatulifolius* Beille. The preparation that has been used in the treatment of bronchial asthma of wind-phlegm obstructing the lung in acute attack period for many years is widely used in Guangxi Zhuang Autonomous Region of China [1]. It has the efficacy of reducing fever and removing toxins, relieving cough and resolving phlegm. Moreover, it has been proven by long-term clinical practice that Zhideke granules have an effect on flu, fever, cough, bronchial asthma, etc. [2, 3]. Although it has been reported that baicalin in *Scutellaria baicalensis* Georgi. and tectoridin and irisflorentin in *Belamcanda chinensis* (L.) Redouté are used as quality control components of Zhideke granules, the material base of it is still unclear. At the same time, some literatures about baicalin [4, 5] and tectoridin [6, 7] proved that they had antiasthma effect and reduced inflammation. However, other chemical constituents and pharmacodynamic substances of Zhideke granules have not been reported.

The ultra-performance liquid chromatography coupled with hybrid quadrupole-orbitrap mass spectrometry (UPLC-Q-Orbitrap HRMS) is a new technology developed in recent years for analyzing the structure of complex traditional Chinese medicine (TCM) and its compound preparations [8–11], and it has the advantages of high efficiency, high speed, high sensitivity, and high resolution and specificity [12–14]. In this study, UPLC-Q-Orbitrap HRMS was used to rapidly isolate and identify unknown chemical components in Zhideke granules for the first time. We analysed the secondary fragment ion information of the target compound as well as relevant literature to further determine the possible chemical constituents in Zhideke granules, which has provided a reference for the quality control and its pharmacodynamics substances of Zhideke granules.

## 2. Materials and Methods

### 2.1. Chemicals and Reagents

Zhideke granules were provided by Ruikang Hospital affiliated to Guangxi University of Chinese Medicine. UPLC-grade acetonitrile was purchased from Merck (Merck, Germany). UPLC-grade ammonium acetate was obtained from Shanghai Sixin Biotechnology Co., Ltd. (Shanghai, China). Formic acid and methanol at UPLC-grade were acquired from Fisher (Fisher, USA). Analytical grade ethyl acetate was purchased from Fisher (Fisher, USA). Ultra-pure water was purified with Milli-Q synergy (Millipore, USA). Forsythoside A (no. 111810-201606), baicalin (no. 110715-201720), and wogonin (no. 111514-201706) were purchased from the National Institutes for Food and Drug Control (Beijing, China). Forsythoside A, baicalin, and wogonin purity were found to be above 97.2%, 93.5%, and 96.3%, respectively. Other chemicals were of analytical grade and their purity was above 99.5%.

### 2.2. Ethyl Acetate Extracts Preparation

A 0.1 g sample of Zhideke granules was weighed by using a XS205DU electronic balance (Mettler-Toledo, Switzerland) and extracted with 20 mL of water in an ultrasonic bath (40 kHz, 500 W) for 30 min. Then, the solutions were extracted twice with 20 mL ethyl acetate and combined two ethyl acetate extracts. Subsequently, these extracts were evaporated at the temperature of 80°C by water bath, and the residue was dissolved in 5 mL of methanol. Finally, the solution of the residue was filtered and was analysed.

### 2.3. The Sample Solution of HPLC Analysis

A 2.0 g sample of Zhideke granule was precisely weighed and extracted with 10 mL of methanol in an ultrasonic bath for 30 min. Then, the extract was centrifuged at 13000 r/min for 10 min by using a TGL-16G centrifuge (Shanghai Anting Scientific Instrument Factory, China) and the supernatant was taken as the sample solution. Finally, the solution was analysed by HPLC.

### 2.4. Standards Preparation

We accurately weighed appropriate amounts of baicalin and wogonin, respectively, and then dissolved with methanol to prepare a mixture solution including two standards. Forsythoside A standard solution was prepared by dissolving 11.84 mg each of accurately weighed pure compound in 5 mL methanol.

### 2.5. HPLC Chromatographic Conditions

Separation was achieved on Agilent ZORBAX Eclipse Plus-C18 column (4.6 × 250 mm, 5 *μ*m). The mobile phase was methanol (*A*) and 0.2% phosphoric acid (*B*) by gradient elution (0–30 min, 5%–20% *A*; 30–50 min, 20%–35% *A*; 50–75 min, 35%–40% *A*; 75–110 min, 40%–60% *A*; 110–150 min, 60%–90% *A*) with photo-diode array (PDA) detection at 254 nm at the flow rate of 0.8 mL/min. The column temperature was 30°C and the volume was 10 *μ*L.

### 2.6. UPLC Chromatographic Conditions

In the UPLC system, the column was a Thermo Hypersil Gold C18 column (2.1 mm × 100 mm, 1.9 *μ*m). The mobile phase consisted of 0.1% formic acid acetonitrile (A) and 0.1% formic acid water containing 10 mmoL of ammonium acetate (B) which was programmed with a gradient elution (0–2.0 min, 5% *A*; 2.0 to 42.0 min, 5% to 95% *A*; 42.0 to 47.0 min, 95% *A*; 47.1 to 50 min, 95% to 5% *A*) at a flow rate of 0.3 mL/min. The sample injection volume was 1 *μ*L. The column temperature was maintained at 35°C.

### 2.7. Instruments and MS Conditions

Chemical constituent's analyses were performed on Thermo Fisher U3000 UPLC system (Thermo Fisher, USA), Trace Finder software (Thermo Fisher, USA), which was used for the UPLC-Q-Exactive Orbitrap MS data processing. The ion source was the heated electrospray ionization (ESI). The electrospray ionization source in both positive and negative ion modes was used in MS analysis. Spray voltages were set at 3.5 kV in a positive ion mode and 3.2 kV in a negative ion mode, respectively. The auxiliary gas temperature was 300°C, and the capillary temperature was 320°C. MS data were obtained on Full MS/dd-MS2 mode in the mass range of 100–1500 Da. The resolution of the precursor mass was 70000 FWHM, while the resolution of the product mass was 17500 FWHM. The specific ion scan mode was off. High purity nitrogen was used as the collision gas, and nitrogen was used as spray gas. The flow rates of sheath gas and auxiliary gas were at the rate of 30 and 10 *μ*L/min, respectively.

## 3. Results and Discussion

**Figure 1:**
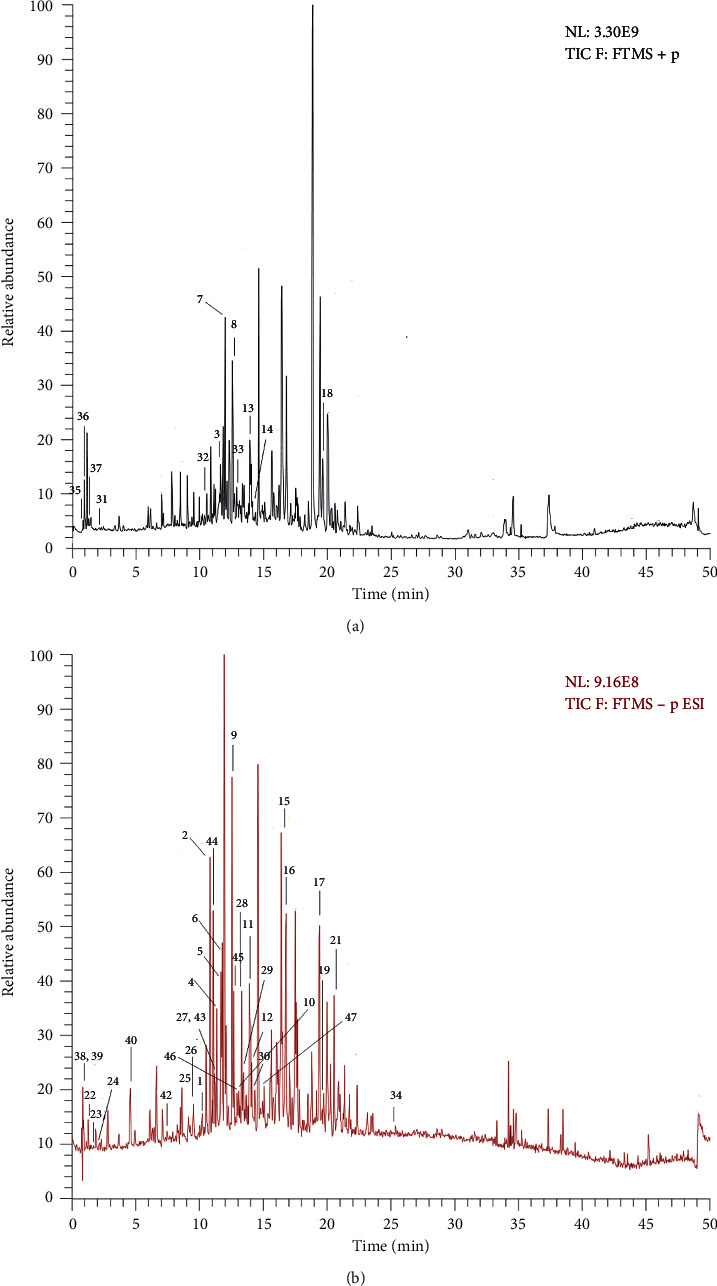
The total ion current chromatogram in positive (a) and negative ions (b). Mode for ethyl acetate of Zhideke granules.

**Figure 2:**
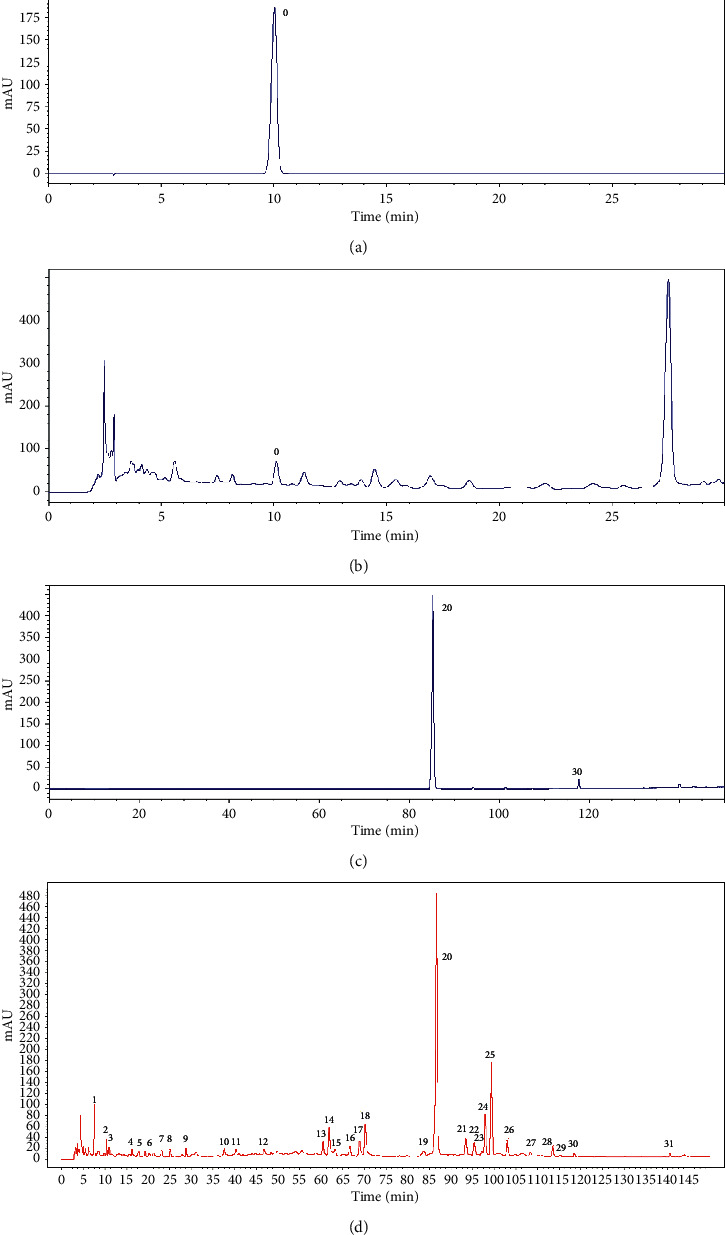
HPLC-UV (PDA) chromatograms obtained from the standard of forsythoside A (a), the sample solution (b), a mixture of two standards (c), and HPLC fingerprint of Zhideke granules (d).

We chose 50% ethyl acetate as the extraction solvent and identified chemical constituents from the ethyl acetate extract of Zhideke granules by UPLC-Q-Orbitrap MS in this paper. In the positive and negative ion modes, the total ion chromatograms of ethyl acetate extract in Zhideke granules are shown in Figure 1. The HPLC spectra of the standard of forsythoside A (A), the sample solution (B), a mixture of two standards (C), and the HPLC fingerprint of Zhideke granules (D) are shown in Figure 2.

According to MS mass, MS/MS fragmentation information, fragmentation patterns, and literature reports, we identified 47 possible chemical constituents including 21 flavonoids and glycosides, 9 organic acids, 4 volatile components, 3 nitrogen-containing compounds, and 10 other compounds from the ethyl acetate extract of Zhideke granules. The retention time, mass spectrometry information, and related literature of identified compounds are shown in Table 1.

### 3.1. Identification of Flavonoids and Glycosides

**Figure 3:**
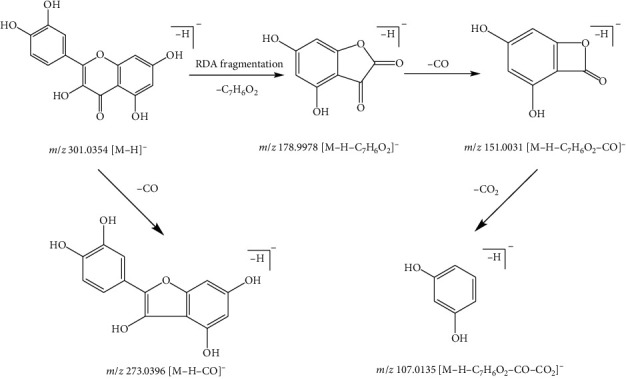
Possible fragmentation pathway of compound 11.

These compounds with a C_6_–C_3_–C_6_ carbon skeleton in the structure called flavonoids had two benzene rings formed by three carbon atoms. The structure of flavonoids often results in substituents such as hydroxyl, methyl, and methoxyl groups. Therefore, in the fragmentation regularity of flavonoids, these compounds easily lose neutral fragments of CO (28 Da), H_2_O (18 Da), CO_2_ (44 Da), and fragment ions of substituents [15]. In addition, the retro-Diels–Alder (RDA) fragmentation is a common fragmentation pattern in flavonoids. Taking compound 11 as an example, compound 11, with the quasimolecular ion *m*/*z* 301.0354 [M-H]^−^ and formula of C_15_H_10_O_7_, was identified as quercetin in the negative ionization mode. The fragment ion at *m*/*z* 273.0396 [M-H-CO]^−^ was derived from the ion at the *m*/*z* 301.0354 by loss of a CO (28 Da) molecule in the MS/MS spectrum. The fragment ion at *m*/*z* 178.9978 [M-H-C_7_H_6_O_2_]^−^ was from the ion at *m*/*z* 301.0354 because of the RDA fragmentation. Subsequently, fragment ions at *m*/*z* 151.0031 [M-H-C_7_H_6_O_2_-CO]^−^ and *m*/*z* 107.0135 [M-H-C_7_H_6_O_2_-CO-CO_2_]^−^ originated from the ion at *m*/*z* 178.9978 by the loss of a CO (28 Da) molecule and a CO_2_ (44 Da) molecule, respectively. Compound 11 was recognized as quercetin and the possible fragment pathway is as shown in Figure 3 according to these results [16]. Compound 17, with the quasimolecular ion *m*/*z* 283.0602 [M-H]^−^ and formula of C_16_H_12_O_5_, was characterized as wogonin by comparing with the reference. Compound 18, with the quasimolecular ion *m*/*z* 403.1387 [M + H]^+^ and formula of C_15_H_10_O_7_, was identified as nobiletin in the positive ionization mode. The ion at *m*/*z* 373.0900 [M + H-2CH_3_]^+^ was from the ion at *m*/*z* 403.1387 by loss of two methyl group fragments. Subsequently, the ion at *m*/*z* 327.0849 [M + H-2CH_3_-H_2_O-CO]^+^ originated from the ion at *m*/*z* 373.0900 by loss of H_2_O (18 Da) and CO (28 Da). Compound 18 was characterized as nobiletin [17] according to these results.

According to the fragmentation process of flavonoids, we presumed that compounds 9, 10, 12, 15, 16, 19, 20, and 21 were identified as scutellarein, eriodictyol, eupafolin, irigenin, baicalein, chrysin, pinocembrin, and tectorigenin, respectively.

**Figure 4:**
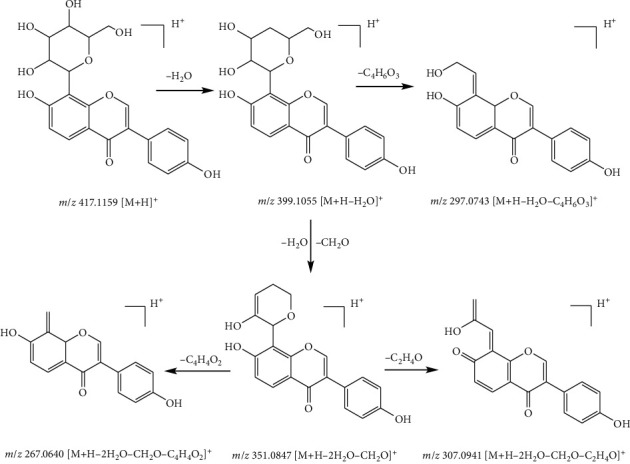
Possible fragmentation pathway of compound 3.

In nature, flavonoids mostly exist in the form of glycosides. Glycosidic bonds easily cleavage in the fragmentation pattern of glycosides. Taking compound 3 as an example with the quasimolecular ion *m*/*z* 417.1159 [M + H]^+^ and formula of C_21_H_20_O_9_, it was identified as puerarin in the positive ionization mode. The ion at *m*/*z* 399.1055 [M + H-H_2_O]^+^ was due to a natural loss of a H_2_O molecule (18 Da). Subsequently, the ion at *m*/*z* 297.07435 [M + H-H_2_O-C_4_H_6_O_3_]^+^ originated from the ion at *m*/*z* 373.0899 by the loss of C_4_H_6_O_3_ (102 Da) fragment. Moreover, the fragment ion at 351.0837 [M + H-2H_2_O-CH_2_O]^+^ came from the ion at *m*/*z* 399.1055 by the loss of a H_2_O molecule (18 Da) and CH_2_O fragment ion (39 Da). Fragment ions at *m*/*z* 267.0640 [M + H-2H_2_O-CH_2_O-C_4_H_4_O_2_]^+^ and *m*/*z* 307.0941 [M + H-2H_2_O-C_2_H_4_O]^+^ were derived from the fragment ion *m*/*z* 351.0837 by the loss of a C_4_H_4_O_2_ (84 Da) fragment and a C_2_H_4_O (44 Da) fragment, respectively. The possible fragmented pathway of compound 3 is shown in Figure 4 according to this fragmental information. Thus, compound 3 was characterized as puerarin [18].

**Figure 5:**
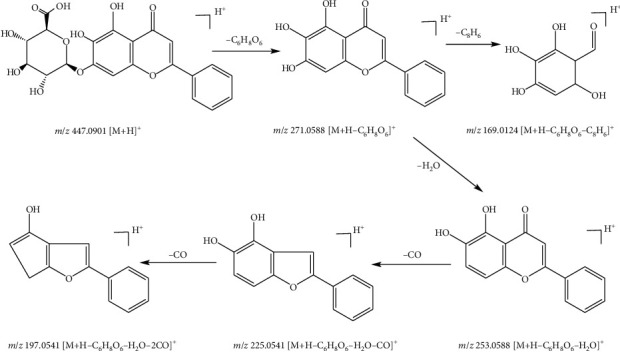
Possible fragmentation pathway of compound 8.

Compound 8, with the quasimolecular ion *m*/*z* 447.0902 [M + H]^+^ and formula of C_21_H_18_O_11_, was identified as baicalin in the positive ion mode. The ion at *m*/*z* 447.0902 produced the ion at *m*/*z* 271.0588 [M + H-C_6_H_8_O_6_]^+^ after the loss of a glucuronic acid molecule (177 Da). The fragmented ion *m*/*z* 169.0124 [M + H-C_6_H_8_O_6_-C_8_H_6_]^+^ was from the ion at *m*/*z* 271.0588 by the loss of a C_8_H_6_ fragment ion (102 Da). Subsequently, the ion at *m*/*z* 225.054 [M + H-C_6_H_8_O_6_-C_8_H_6_-H_2_O-CO]^+^ originates from the ion at *m*/*z* 271.0588 by loss of a H_2_O molecule (18 Da) and a CO molecule (28 Da). The possible fragment pathway of compound 8 is shown in Figure 5 according to this fragmental information. Compound 8 was baicalin by comparing with the reference standard and the literature report [15].

Based on the fragmentation patterns of glycosides, compounds 1, 2, 4, 5, 6, 7, 13, 14, and 20 were possibly identified as isoquercitrin, tectoridin, diosmin, hesperidin, luteolin 7-glucuronide, iridin, linarin, didymin, and pnocembrin, respectively.

### 3.2. Identification of Organic Acids

Organic acids of Zhideke granules are mainly found in *Eriobotrya japonica* (Thunb.), *Platycodon grandiflorus* (Jacq.) A. DC., *Mentha haplocalyx* Briq., etc. Organic acids mainly include two types, one of which is fatty acid and the other is aromatic acid. Organic acids easily lose neutral fragments of CO (28 Da), H_2_O (18 Da), CO_2_ (44 Da), and a fragment of alkyl. Taking compound 24 as an example, compound 24, with the quasimolecular ion *m*/*z* 197.0448 [M-H]^−^ and formula of C_9_H_10_O_5_, was identified as danshensu under the negative ion mode. The fragment ion was at *m*/*z* 179.0342 [M-H-H_2_O]^−^ at the loss of a H_2_O molecule. Fragment ions at *m*/*z* 151.0401 [M-H-H_2_O-CO]^−^ and *m*/*z* 134.0369 [M-H-H_2_O-CO_2_]^−^ were derived from the fragment ion *m*/*z* 179.0343 by the loss of a CO (28 Da) molecule and a CO_2_ (44 Da) molecule, respectively. Based on fragment rules and literature, compound 24 was characterized as danshensu [19].

**Figure 6:**
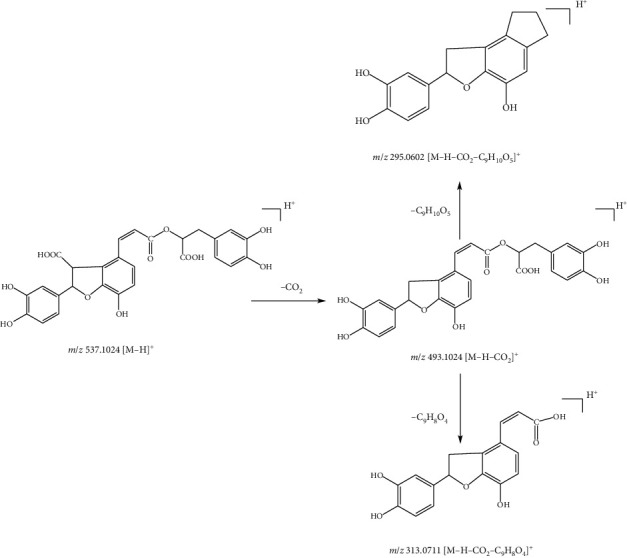
Possible fragmentation pathway of compound 26.

Compound 26, with the quasimolecular ion at *m*/*z* 537.1024 [M-H]^−^ and formula of C_27_H_22_O_12_, was identified as lithospermic acid under the negative ion mode. The fragment ion at *m*/*z* 493.1024 [M-H]^−^ was formed due to the loss of a CO_2_ (28 Da) molecule of the ion at *m*/*z* 537.1024. Fragment ions at *m*/*z* 295.0602 [M-H-CO_2_-C_9_H_10_O_5_]^−^ and *m*/*z* 313.0711 [M-H-C_9_H_8_O_4_]^−^ were derived from the ion at *m*/*z* 493.1027 by the loss of fragment ions of C_9_H_10_O_5_ (198 Da) and C_9_H_8_O_4_ (180 Da). According to the results, compound 26 was identified as lithospermic acid [20]. The fragmentation pathway is shown in Figure 6.

Compound 27, with the quasimolecular ion at *m*/*z* 187.0968 [M-H]^−^ and formula of C_9_H_16_O_4_, was identified as azelaic acid under the negative ion mode. The fragment ion at *m*/*z* 143.1070 [M-H-CO_2_]^−^ was formed due to the loss of a CO_2_ (28 Da) molecule of the ion at *m*/*z* 187.0968. The fragment ion at *m*/*z* 125.0967 [M-H-CO_2_-H_2_O]^−^ was derived from the ion at *m*/*z* 143.1070 by the loss of an H_2_O (18 Da) molecule. Subsequently, the fragment ions at *m*/*z* 97.0654 [M-H-CO_2_-H_2_O-C_2_H_4_]^−^, *m*/*z* 83.0501 [M-H-CO_2_-H_2_O-C_3_H_6_]^−^, and *m*/*z* 69.0342 [M-H-CO_2_-H_2_O-C_4_H_8_]^−^ were derived from the ion at *m*/*z* 125.0967 by the loss of fragment ions of C_2_H_4_ (28 Da), C_3_H_6_ (42 Da), and C_4_H_8_ (56), respectively. Based on MS data and related literature, compound 27 was identified as azelaic acid [21]. According to the fragmentation process [16], compound 29 was determined as abscisic acid.

**Figure 7:**
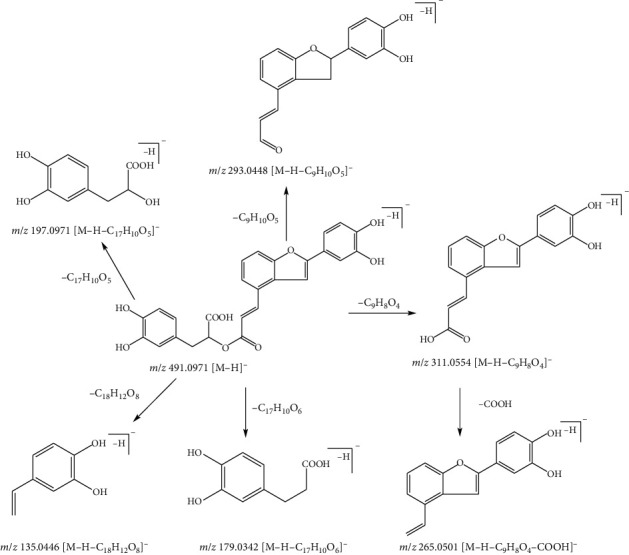
Possible fragmentation pathway of compound 30.

Compound 30, with the quasimolecular ion at *m*/*z* 491.0971[M-H]^−^ and formula of C_26_H_20_O_10_, was identified as salvianolic acid C under the negative ion mode. The fragment ions at *m*/*z* 135.0446 [M-H-C_18_H_12_O_8_]^−^, 179.0342[M-H-C_17_H_12_O_6_]^−^, *m*/*z* 197.0971 [M-H-C_17_H_10_O_5_]^−^, and *m*/*z* 311.0554 [M-H-C_9_H_8_O_4_]^−^ were from the ion at *m*/*z* 491.0970 by the loss of fragment ions C_18_H_12_O_8_ (356 Da), C_17_H_12_O_6_ (312 Da), C_17_H_10_O_5_ (294 Da), and C_9_H_8_O_4_ (180 Da), respectively. Subsequently, the fragment ion at *m*/*z* 265.0501 [M-H-C_9_H_8_O_4_-COOH]^−^ was made up of the ion at *m*/*z* 311.0554 by the loss of fragment ions of the carboxyl group fragment. The ion at *m*/*z* 293.0448 [M-H-C_9_H_10_O_5_]^−^ was from the cleavage of the ion at *m*/*z* 491.0970 occurring in the position of the C-O bond in the ester bond. Therefore, compound 30 was identified as salvianolic acid C by referring to the literature [22]. The fragmentation pathway of salvianolic acid C is shown in Figure 7. Based on fragment rules and literature [22], compound 28 was salvianolic acid A.

Based on the fragmentation rules of organic acids, compounds 22, 23, and 25 were identified as gallic acid, pyrogallol, and p-hydroxycinnamic acid, respectively.

### 3.3. Identification of Volatile Components

Volatile components are mainly found in *Belamcanda chinensis* (L.) Redouté, *Cynanchum glaucescens* (Decne.) Hand.-Mazz., and *Bupleurum chinense* in Zhideke granules. Taking compound 31 as an example, compound 31, with the quasimolecular ion at *m*/*z* 127.0385 [M + H]^+^ and formula of C_6_H_6_O_3_, was identified as 5-hydroxymethylfurfural in the positive ion mode. The fragment ion at *m*/*z* 127.0385 lost the CH_2_OH group (31 Da) and produced the fragment ion at *m*/*z* 97.0285 [M + H-CH_2_OH]^+^. The fragment ion at *m*/*z* 81.0337[M + H-H_2_O-CO]^+^ was derived from the ion at *m*/*z* 127.0385 by the loss of a H_2_O (18 Da) molecule and a CO (28 Da) molecule, successively. Then, the fragment ion at *m*/*z* 81.0337 continuously lost a CO (28 Da) molecule and yielded the fragment ion at *m*/*z* 53.0392 [M + H-H_2_O-2CO]^+^. Based on MS data and relevant literature [23], compound 31 was identified as 5-hydroxymethylfurfural.

Compound 32 showed *m*/*z* 153.1267 [M + H]^+^ ion and a formula of C_10_H_16_O in the positive ion mode of the first-order mass spectrum. In the MS/MS spectrum, we observed fragment ions at *m*/*z* 59.0496, 69.0701, 93.0699, 95.0854, 97.0646, 107.0854, 109.1008, and 135.1165. These results of fragmentation patterns are consistent with the relevant literature [24]. Thus, compound 32 was identified as camphor.

**Figure 8:**
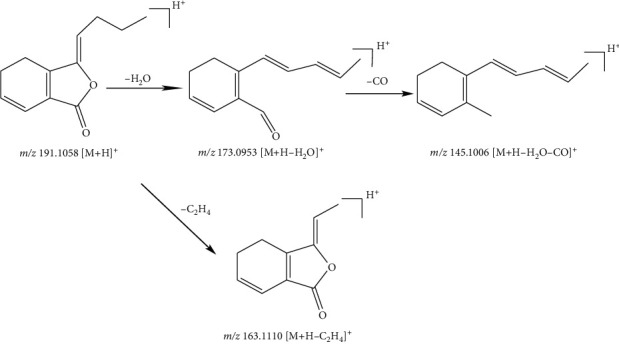
Possible fragmentation pathway of compound 33.

Compound 33, with the quasimolecular ion at *m*/*z* 191.1058 [M + H]^+^ and formula of C_12_H_14_O, was identified as ligustilide in the positive ion mode. Fragment ions at *m*/*z* 163.1110 [M + H-C_2_H_4_]^+^ and *m*/*z* 173.0953 [M + H-H_2_O]^+^ were from the ion at *m*/*z* 191.1058 by the loss of C_2_H_4_ (28 Da) fragment and an H_2_O (18 Da) molecule, respectively. Subsequently, the fragment ion at *m*/*z* 173.0953 [M + H-H_2_O]^+^ was derived from the ion at *m*/*z* 173.0953 by the loss of a CO (28 Da) molecule. According to relevant literature [25], compound 33 was identified as ligustilide. The fragmentation pathway of compound 33 is given in Figure 8.

Compound 34, with the quasimolecular ion at *m*/*z* 315.2528 [M + HCO_2_]^−^ and formula of C_17_H_34_O_2_, was identified as methyl palmitate in the positive ion mode. Fragment ions at *m*/*z* 127.1122 C_9_H_19_^+^, *m*/*z* 141.1279 C_10_H_21_^+^, were formed because of cleavage of the alkyl group of the ester compounds. In addition, the fragment ion at *m*/*z* 171.1020 [M-H-C_7_H_15_]^−^ was derived from the [M-H]^−^ ion. Thus, compound 34 was identified as methyl palmitate based on the fragmentation rules and related reports [23].

### 3.4. Identification of Nitrogen-Containing Compounds

Nitrogen-containing compounds refer to a class of organic compounds containing a nitrogen element in the structure of the molecule and mainly include a nucleoside, an amino acid, and nicotinamide. The nitrogen-containing compounds are mainly derived from *Mentha haplocalyx* Briq. and *Bupleurum chinense* in Zhideke granules. The natural loss of H_2_O (18 Da), CO_2_ (44 Da), and NH_3_ (17 Da) molecules easily takes place in the fragmentation process of nitrogen-containing compounds. Besides, amino acid molecules easily lost the carboxyl group (45 Da) and a hydroxyl group (17 Da).

Compound 35, with the quasimolecule ion at *m*/*z* 195.0867 [M + H]^+^ and formula of C_8_H_10_N_4_O_2_, was identified as caffeine in the positive ion mode. The fragment ions at *m*/*z* 138.0656 [M + H-C_2_H_4_N_2_]^+^, *m*/*z* 110.0711 [M + H-C_4_H_10_N_2_]^+^, *m*/*z* 69.0451 [M + H-C_5_H_10_N_3_O]^+^, and *m*/*z* 56.0500 [M + H-C_6_H_6_N_2_O_2_]^+^ were derived from the fragment ion at *m*/*z* 195.0867 by the loss of the fragment ions of C_2_H_4_N_2_ (56 Da), C_4_H_10_N_2_ (86 Da), C_5_H_10_N_3_O (128 Da), and C_6_H_6_N_2_O_2_ (138 Da), respectively. The fragmentation patterns are basically consistent with the literature [26]. Thus, compound 35 was identified as caffeine. According to the fragmentation pattern, compound 36 was identified as adenine [27].

Compound 37, with the quasimolecule ion at *m*/*z* 123.0548[M + H]^+^ and formula of C_6_H_6_N_2_O, was identified as nicotinamide in the positive ion mode. The fragment ions at 105.0445 [M + H-NH_3_]^+^ and *m*/*z* 95.0606 [M + H-CO]^+^ were from the ion at *m*/*z* 123.0548 by the loss of a NH_3_ (17 Da) molecule fragment and a CO (28 Da) molecule, respectively. Besides, the fragment ion at *m*/*z* 80.0497 [M + H-CO-NH_2_]^+^ was formed owing to the loss of the NH_2_ (16 Da) fragment. Therefore, compound 37 was identified as nicotinamide [28].

### 3.5. Identification of Other Compounds

**Figure 9:**
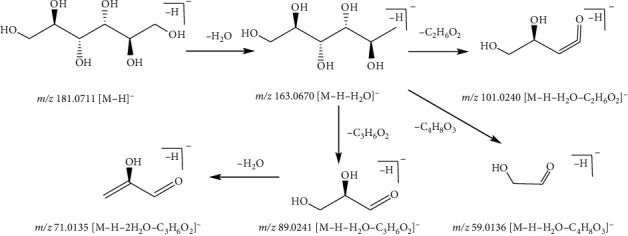
Possible fragmentation pathway of compound 38.

There were some other compounds in the Zhideke granules, except constituents mentioned in the previous discussion such as sugar, coumarin, and phenol. Taking 38 as an example, compound 38, with the quasimolecular ion at *m*/*z* 181.0711 [M + H]^+^ and a formula of C_6_H_14_O_6_, was identified as mannitol in the positive ion mode. The fragment ion at *m*/*z* 163.0670 [M-H-H_2_O]^−^ was due to the loss of a H_2_O (18 Da) molecule of the ion at *m*/*z* 181.0711. The fragment ions at *m*/*z* 101.0240 [M-H-H_2_O-C_2_H_6_O_2_]^−^, *m*/*z* 59.0136 [M-H-H_2_O-C_4_H_8_O_3_]^−^, and *m*/*z*89.0241 [M-H-H_2_O-C_3_H_6_O_2_]^−^ were formed from the fragment ion at *m*/*z* 163.0670 by the loss of the fragment ions of C_2_H_6_O_2_ (62 Da), C_4_H_8_O_3_ (104 Da), and C_3_H_6_O_2_ (74 Da), respectively. Subsequently, the fragment ion at *m*/*z* 89.0241 [M-H-H_2_O-C_3_H_6_O_2_]^−^ lost a H_2_O (18 Da) molecule and yielded *m*/*z* 71.0135 [M-H-2H_2_O-C_3_H_6_O_2_]^−^. Taking the literature into account [29], compound 38 was identified as mannitol. The fragmentation pathway of mannitol is shown in Figure 9.

Compound 39 showed *m*/*z* 341.1078 [M-H]^−^ ion and a formula of C_12_H_22_O_11_ in the negative ion mode of the first-order mass spectrum. In the MS/MS spectrum, the fragment ions at *m*/*z* 59.0136, 71.0136, 89.0241, 95.0137, and 113.0241 were obtained. These fragmentation patterns were consistent with the literature report [25]. Therefore, compound 39 was recognized as sucrose.

Compound 40, with the quasimolecular ion at *m*/*z* 137.0238 [M-H]^−^ and formula of C_7_H_6_O_3_, was identified as protocatechualdehyde in the negative ion mode. The characteristic fragment ion at *m*/*z* 93.0341 [M-H-CO_2_]^−^ originates from the ion at *m*/*z* 137.0238 by the loss of a CO_2_ (44 Da) molecule. Based on the reported literature [30], compound 40 was identified as protocatechualdehyde.

Compound 41, with the quasimolecular ion at *m*/*z* 177.0186 [M-H]^−^ and formula of C_9_H_6_O_4_, was identified as esculetin in the negative ion mode. Fragment ions at *m*/*z* 133.0290 [M-H-CO2]^−^ and *m*/*z* 149.0237 [M-H-CO]^−^ were derived from the fragment ion at *m*/*z* 177.0186 by the loss of a CO_2_ (44 Da) molecule and a CO (28 Da) molecule, respectively. Thus, compound 41 was identified as esculetin [31]. Meanwhile, compound 45 was identified as 5,7-dihydroxy-4-methyl coumarin according to the fragmentation pattern [32].

Compound 42, with the quasimolecular ion at *m*/*z* 421.0766 [M-H]^−^ and formula of C_19_H_18_O_11_, was identified as isomangiferin in the negative ion mode. The fragment ion at 258.0161 [M-H-C_6_H_10_O_5_]^−^ was formed from the cleavage of the ion at *m*/*z* 421.0766 occurring in the position of the glucoside bond by the loss of the C_6_H_10_O_5_ (162 Da) fragment ion. Therefore, compound 42 was identified as isomangiferin [33].

Compound 43, with the quasimolecular ion at *m*/*z* 243.0655 [M-H]^−^ and formula of C_14_H_12_O_4_, was identified as piceatannol in the negative ion mode. The fragment ions at *m*/*z* 225.0549 [M-H-H_2_O]^−^, *m*/*z* 159.0445 [M-H-C_4_H_4_O_2_], and *m*/*z* 201.0549 [M-H-C_2_H_2_O]^−^ originated from the ion at *m*/*z* 243.0655 by the loss of a H_2_O (18 Da) molecule, a C_4_H_4_O_2_ (84 Da) fragment, and a C_2_H_2_O (42 Da) fragment. Subsequently, the fragment ion *m*/*z* 173.0601 [M-H-C_2_H_2_O-CO]^−^ was formed from the ion at *m*/*z* 201.0549 [M-H-C_2_H_2_O]^−^ due to a CO (28 Da) molecule. Compound 43 was identified as piceatannol [34].

Compound 44 showed *m*/*z* 623.1963[M-H]^−^ ion and a formula of C_29_H_36_O_15_ in the negative ion mode of the first-order mass spectrum. Fragment ions at *m*/*z* 461.1656 [M-H-C_9_H_6_O_3_]^−^ and *m*/*z* 179.0341 [M-H-C_20_H_28_O_11_]^−^ were derived from the fragment ion at *m*/*z* 623.1966 by the loss of the fragments C_9_H_6_O_3_ (162 Da) and C_20_H_28_O_11_ (444 Da), respectively. Furthermore, the fragment ion at *m*/*z* 179.0341 lost a H_2_O (18 Da) molecule and yielded the fragment ion at *m*/*z* 161.0238 [M-H-C_20_H_28_O_11_-H_2_O]^−^. Based on relevant literature [35], compound 44 may be forsythoside A, isoacteoside, or verbascoside. Then, compound 44 was identified as forsythoside A by comparing with the reference standard.

**Figure 10:**
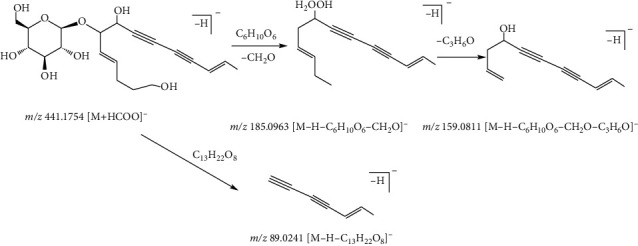
Possible fragmentation pathway of compound 46.

Compound 46, with the quasimolecular ion at *m*/*z* 441.1754 [M + HCOOH]^−^ and formula of C_20_H_28_O_8_, was identified as lobetyolin in the negative ion mode. The fragment ion at *m*/*z* 185.0963 [M-H-C_6_H_10_O_6_-CH_2_O]^−^ was derived from the ion at *m*/*z* 441.1754 by the loss of a fragment of the C_6_H_10_O_6_ (178 Da) and CH_2_O (30 Da) groups. Subsequently, the fragment ion at *m*/*z* 159.0811 [M-H-C_6_H_10_O_6_-CH_2_O-C_3_H_6_O]^−^ originated from the ion at *m*/*z* 185.0963 by the loss of C_3_H_6_O (58 Da) fragment. In addition, the fragment ion at *m*/*z* 89.0241 [M-C_13_H_22_O_8_]^−^ was formed from the ion at *m*/*z* 441.1754 by the loss of C_13_H_22_O_8_ fragment. Thus, compound 46 was identified as lobetyolin [36]. The fragmentation pathway of lobetyolin is shown in Figure 10.

**Figure 11:**
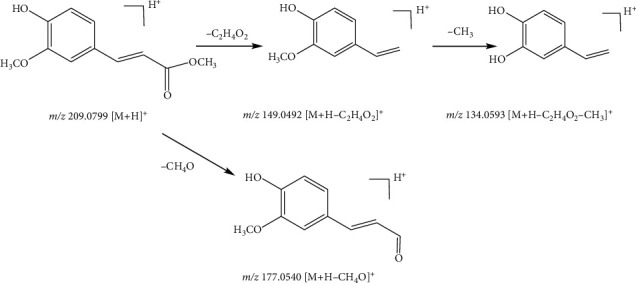
Possible fragmentation pathway of compound 47.

Compound 47, with the quasimolecular ion at *m*/*z* 209.0799 [M + H]^+^ and formula of C_11_H_12_O_4_, was identified as methyl 4-hydroxy-3-methoxycinnamate in the positive ion mode. Fragment ions at *m*/*z* 177.0540 [M-H-CH_4_O]^+^ and *m*/*z* 149.0492 [M-H-C_2_H_4_O_2_]^+^ were derived from the ion at *m*/*z* 209.0799 by the loss of fragments of CH_4_O (32 Da) and C_2_H_4_O_2_ (60 Da). Subsequently, the fragment ion at *m*/*z* 134.0593 [M-H-C_2_H_4_O-CH_3_]^+^ was formed from the ion at *m*/*z* 149.0492 by loss of the CH_3_ (15 Da) fragment. Therefore, compound 47 was identified as ferulic acid methyl ester [12]. The fragmentation pathway of ferulic acid methyl ester is shown in Figure 11.

## 4. Conclusions

The paper provided a new analysis method for the chemical constituents research of Zhideke granules. At the same time, this study rapidly and systematically analysed the chemical constituents of Zhideke granules by UPLC coupled with hybrid quadrupole-orbitrap MS. 47 chemical constituents were identified by UPLC-Q-Orbitrap HRMS including flavonoids and glycosides, organic acids, volatile components, nitrogen-containing compounds, sugars, and coumarins, which will provide a reference for quality control of Zhideke granules, and further reveal the pharmacodynamic substances of Zhideke granules.

## Figures and Tables

**Table 1:** Characterization of chemical constituents from the ethyl acetate extract of Zhideke granules by UPLC coupled with hybrid quadrupole-orbitrap MS.

Type of compounds	No.	*tR* (min)	Adduct ions	Theoretical (*m*/*z*)	Measured (*m*/*z*)	Mass error (ppm)	Molecular formula	MS/MS (*m*/*z*)	Identification compounds	Source
Flavonoids and glycosides	1	10.21	M-H	463.0882	463.087	−2.5133	C_21_H_20_O_12_	255.0291 [M^−^H-Glc-O-CHO]^−^ 271.0241 [M-Glc-CHO]^−^	Isoquercitrin	*Bupleurum chinense*, *Eriobotrya japonica* (Thunb.), *Sauropus spatulifolius* Beille. [37]
	2	10.82	M + HCO_2_	461.1089	461.1078	−2.5426	C_22_H2_2_O_11_	298.0472 [M-H-C_6_H_10_O_5_]^−^ 284.0317 [M-H-C_6_H_10_O_5_-CH_3_]^−^	Tectoridin	*Belamcanda chinensis* (L.) Redouté [32]
	3	11.34	M + H	417.118	417.1159	−4.9715	C_21_H_20_O_9_	267.0640 [M + H-2H_2_O-CH_2_O-C_4_H_4_O_2_]^+^ 297.0743 [M + H-C_4_H_8_O_4_]^+^ 307.0941 [M + H-2H_2_O-C_2_H_4_O]^+^ 351.0847 [M + H-2H_2_O-CH_2_O]^+^ 399.1055 [M + H-H2O]^+^	Puerarin	*Bupleurum chinens*e [18]
	4	11.65	M-H	607.1668	607.1653	−2.5640	C_28_H_32_O_15_	299.0550 [M-H-C_12_H_21_O_9_]^−^	Diosmin	*Mentha haplocalyx* Briq. [38]
	5	11.75	M-H	609.1825	609.1812	−2.1958	C_28_H_34_O_15_	301.0707 [M-H-rutinoses]^−^	Hesperidin	*Eriobotrya japonica* (Thunb.), *Mentha haplocalyx* Briq., *Nepeta cataria* L., *Nervilia fordii* (Hance) Schltr. [15]
	6	11.82	M-H	461.0726	461.0714	−2.6024	C_21_H_18_O_12_	285.0395 [M-H-C_6_H_8_O_6_]^−^	Luteolin 7-glucuronide	*Mentha haplocalyx* Briq. [39]
	7	11.95	M + H	523.1446	523.1423	−4.4864	C_24_H_26_O_13_	361.0899 [M + H-C_6_H_10_O_5_	Iridin	*Belamcanda chinensis* (L.) Redouté [33, 40]
	8^a^	12.52	M + H	447.0922	447.0901	−4.6335	C_21_H_18_O_11_	169.0124 [M + H-C6H8O6-C_8_H_6_]^+^ 225.0541 [M + H-C6H8O6-H2O-CO]^+^ 271.0588 [M + H-C6H8O6]^+^	Baicalin	*Scutellaria baicalensis* Georgi.， *Bupleurum chinense* [15]
	9	12.58	M-H	285.0405	285.0395	−3.511	C_15_H_10_O_6_	165.0185 [M-H-C8H8O]^+^ 239.0343 [M-H-H2O-CO]^+^	Scutellarein	*Scutellaria baicalensis* Georgi. [41]
	10	12.83	M-H	287.0561	287.0552	−3.0419	C_15_H_12_O_6_	267.0290 [M-H-H2O]^+^ 135.0446 [M-H-C7H4O4]^−^	Eriodictyol	Unknown [24]
	11	13.91	M-H	301.0354	301.0343	−3.6832	C_15_H_10_O_7_	107.0135 [M-H-C_7_H_6_O_2_-CO-CO_2_]^−^ 151.0031 [M-H-C7H6O2-CO]^−^ 178.9978 [M-H-C_7_H_6_O_2_]^−^ 273.0396 [M-H-CO]^−^	Quercetin	*Nepeta cataria* L., *Bupleurum chinense*, *Eriobotrya japonica* (Thunb.), *Sauropus spatulifolius* Beille. [16]
	12	13.94	M-H	315.051	315.05	−3.1143	C_16_H_12_O_7_	300.0175 [M-H-CH_3_]^−^	Eupafolin	*Scutellaria baicalensis* Georgi. [42]
	13	14.10	M + H	593.1865	593.1837	−4.7628	C_28_H_32_O_14_	242.0559M + H-C_12_H_20_O_9_-CH_3_-CO]^+^ 285.0742 [M + H-C12H20O9]^+^ 447.1261 [M + H-C6H10O4]^+^	Linarin	*Mentha haplocalyx* Briq. [43]
	14	14.36	M + H	595.2021	595.1993	−4.7887	C_28_H_34_O_14_	287.0900 [M + H-Rha- Glc]^+^	Didymin	*Mentha haplocalyx* Briq. [15]
	15	16.39	M-H	359.0772	359.0761	−3.1446	C_18_H_16_O_8_	301.0347 [M-H-2CH_3_- CO]^−^	Irigenin	*Belamcanda chinensis* (L.) Redouté [33]
	16	16.78	M-H	269.0456	269.0446	−3.4689	C_15_H_10_O_5_	223.0594 [M-H-H_2_O-CO]^−^	Baicalein	*Scutellaria baicalensis* Georgi. [41]
	17	19.42	M-H	283.0612	283.0602	−3.6011	C_16_H_12_O_5_	240.0426 [M + H-CH3- CO]^−^	Wogonin	*Scutellaria baicalensis* Georgi. [44, 45]
	18	19.54	M + H	403.1387	403.1368	−4.8624	C_21_H_22_O_8_	327.0849 [M + H-2CH3- H2O-CO]^+^ 373.0900 [M + H-2CH3]^+^ 119.0497 [M-H-C3O2- C4H2O]^−^	Nobiletin	Unknown [17]
	19	19.74	M-H	253.0506	253.0498	−3.1409	C_15_H_10_O_4_	143.0495 [M-H-C_3_O_2_- C_2_H_2_O]^−^ 181.0652 [M-H-CO2- CO]^−^ 209.0599 [M-H-CO_2_]^−^ 107.0133 [M-H-C_8_H_8_- CO_2_]^−^	Chrysin	*Mentha haplocalyx* Briq., *Scutellaria baicalensis* Georgi. [46]
	20	20.1 4	M-H	255.0663	255.0655	−2.9149	C_15_H_12_O_4_	145.0652 [M-H-C_3_O_2_- C_2_H_2_O]^−^ 171.0448 [M-H- 2C_2_H_2_O]^−^ 211.0753 [M-H-CO_2_]^−^	Pinocembrin	Unknown [46]

Organic acids	21	20.54	M-H	299.0561	299.0551	−3.5321	C_16_H_12_O_6_	227.0344 [M-H-CO2^−^CO]^−^ 255.0292 [M-H-CO_2_]^−^	Tectorigenin	*Belamcanda chinensis* (L.) Redouté [33]
	22	1.50	M-H	169.0142	169.0136	−3.7817	C_7_H_6_O_5_	69.0343 [M-H-C_3_O_4_]^−^ 97.0291 [M-H-C_2_O_3_]^−^ 125.0239 [M-H-CO]^−^	Gallic acid	*Eriobotrya japonica* (Thunb.) [16, 47]
	23	1.86	M-H	125.0244	125.0239	−3.892	C_6_H_6_O_3_	81.0187 [M-H-H2O-CO]^−^	Pyrogallol	Unknown [48]
	24	2.19	M-H	197.0456	197.0448	−3.8072	C_9_H_10_O_5_	134.0369 [M-H-H_2_O-CO_2_]^−^ 151.0401 [M-H-H_2_O-CO]^−^ 179.0342 [M-H-H_2_O]-	Danshensu	*Mentha haplocalyx* Briq. [19]
	25	8.62	M^−^H	163.0401	163.0395	−3.4654	C_9_H_8_O_3_	119.0497 [M-H-CO_2_]^−^	p-Hydroxy-cinnamic acid	Unknown [37]
	26	9.85	M-H	537.1038	537.1024	−2.7817	C_27_H_22_O_12_	295.0602 [M-H-CO_2_-C_9_H_10_O_5_]^−^ 313.0711 [M-H-C_9_H_8_O_4_]^−^	Lithospermic acid	*Mentha haplocalyx* Briq. [20]
	27	11.68	M-H	187.0976	187.0968	−4.1596	C_9_H_16_O_4_	69.0342 [M-H-CO_2_-H_2_O-C_4_H_8_]^−^ 83.0501 [M-H-CO_2_-H_2_O-C_3_H_6_]^−^ 97.0654 [M-H-CO_2_-H_2_O-C_2_H_4_]^−^ 125.0967 [M-H-CO_2_-H2O]^−^ 143.1070 [M-H-CO_2_]^−^	Azelaic acid	*Cynanchum glaucescens* (Decne.) Hand-Mazz [21]

Volatile components	28	13.37	M-H	493.114	493.1129	−2.1789	C_26_H_22_O_10_	185.0238295.0601 [M-H-C_15_O_7_H_16_]^−^ 295.0601 [M-H-C_9_O_5_H_1O_]^−^ 313.0709 [M-H-C_9_O_4_H_8_]^−^	Salvianolic acid A	*Mentha haplocalyx* Briq. [22]
	29	13.47	M-H	263.1289	263.128	−3.2636	C_15_H_20_O_4_	204.1147 [M-H-CO_2_-CH_3_]	Abscisic acid	Unknown [16]
	30	14.16	M-H	491.0984	491.0971	−2.6341	C_26_H_20_O_10_	135.0446 [M-H-C_18_H_12_O_8_]^−^ 179.0342 [M-H-C_17_H_12_O_6_]^−^ 197.0971 [M-H-C_17_H_10_O_5_]^−^ 265.0501 [M-H-C9H8O4-COOH]^−^ 293.0448 [M-H-C_9_H_10_O_5_]^−^ 311.0554 [M-H-C_9_H_8_O_4_]^−^	Salvianolic acid C	*Mentha haplocalyx* Briq. [22]
	31	2.17	M + H	127.039	127.0385	−3.5959	C_6_H_6_O_3_	53.0392 [M + H-H2O-2CO]^+^ 81.0337 [M + H-H2O-CO]^+^ 97.0285 [M + H-CH2OH]^+^	5-Hydroxymethylfurfural	*Belamcanda chinensis* (L.) Redouté, *Cynanchum glaucescens* (Decne.) Hand-Mazz. [49]
	32	10.98	M + H	153.1274	153.1267	−4.2481	C_10_H_16_O	135.1165 [M + H-H2O]^+^	Camphor	*Eriobotrya japonica* (Thunb.), *Bupleurum chinense*, *Scutellaria baicalensis* Georgi., *Mentha haplocalyx* Briq., *Cynanchum glaucescens* (Decne.) Hand-Mazz. [24]

Nitrogen-containing compounds	33	12.95	M + H	191.1067	191.1058	−4.3172	C_12_H_14_O_2_	145.1006 [M + H-H2O-CO]^+^ 163.1110 [M + H-C2H4]^+^ 173.0953 [M + H-H2O]^+^	Ligustilide	*Bupleurum chinense* [25]
	34	25.35	M + HCO_2_	315.2541	315.2528	−3.9393	C17H34O2	127.1122 [M-H-C8H15O2]^−^ 141.1279 [M-H-C7H13O2]^−^ 171.1020 [M-H-C7H15]^−^	Methyl palmitate	*Scutellaria baicalensis* Georgi., *Bupleurum chinense*, *Belamcanda chinensis* (L.) Redouté [23]
	35	0.32	M + H	195.0876	195.0867	-4.7109	C_8_H_10_N_4_O_2_	56.0500 [M + H-C_6_H_6_N_2_O_2_]^+^ 69.0451 [M + H-C5H10N3O]^+^ 110.0711 [M + H-C_4_H_10_N_2_]^+^ 138.0656 [M + H-C_2_H_4_N_2_]^+^	Caffeine	Unknown [36]
	36	0.90	M + H	136.0618	136.0612	−4.5036	C_5_H_5_N_5_	92.0244 [M-CH_3_N_2_] 109.0507 [M-CN]^+^	Adenine	Unknown [27]
	37	1.26	M + H	123.0553	123.0549	−3.2842	C_6_H_6_N_2_O	80.0497 [M + H-CO-NH_2_]^+^ 95.0605 [M + H-CO]^+^ 105.0449 [M + H-NH3]^+^	Nicotinamide	*Sauropus spatulifolius* Beille. [28]

Other compounds	38	0.85	M-H	181.0718	181.0711	−3.7806	C_6_H_14_O_6_	59.0136 [M-H-H_2_O-C4H8O3]^−^ 71.0135 [M-H-2H2O-aC3H6O2]^−^ 89.0241 [M-H-H_2_O-C3H_6_O_2_]^−^ 101.0240 [M-H-H_2_O-C_2_H_6_O_2_]^−^ 163.0604 [M-H-H_2_O]^−^	Mannitol	*Sauropus spatulifolius* Beille. [29]
	39	0.85	M-H	341.1089	341.1078	−3.4371	C_12_H_22_O_11_	71.0135 [M-H-C_9_H_16_O_8_-H_2_O]^−^ 89.0241 [M-H-C_9_H_16_O_8_]^−^	Sucrose	*Cynanchum glaucescens* (Decne.) Hand-Mazz. [25]
	40	4.56	M-H	137.0244	137.0238	−4.2749	C_7_H_6_O_3_	93.0341 [M-H-CO_2_]^−^	Protocatechualdehyde	*Mentha haplocalyx* Briq., *Nepeta cataria* L. [30]
	41	6.30	M-H	177.0193	177.0186	−4.0350	C_9_H_6_O_4_	133.0290 [M-H-CO_2_]^−^ 149.0237 [M-H-CO]^−^	Esculetin	*Bupleurum chinense, Nervilia fordii* (Hance) Schltr. [31]
	42	7.50	M-H	421.0776	421.0766	−2.5207	C_19_H_18_O_11_	258.0161 [M-H-C_6_H_10_O_5_]^−^	Isomangiferin	*Belamcanda chinensis* (L.) Redouté [33]
	43	10.61	M-H	243.0663	243.0655	−3.3727	C_14_H_12_O_4_	173.0601 [M-H-C_2_H_2_O-CO]^−^ 201.0549 [M-H-C_2_H_2_O]^−^ 225.0549 [M-H-H_2_O]^−^	Piceatannol	Unknown [34]
	44 a	11.07	M-H	623.1981	623.1963	−2.9701	C_29_H_36_O_15_	161.0238 [M-H-C_20_H_28_O_11_-H_2_O]^−^ 179.0341 [M-H-C_20_H_28_O_11_]^−^ 461.1656 [M-H-C_9_H_6_O_3_]^−^	Forsythoside A	*Cynanchum glaucescens* (Decne.) Hand-Mazz. [35]
	45	12.22	M-H	191.035	191.0343	−3.3107	C_10_H_8_O_4_	163.0393 [M-H-CO]^−^	5,7-Dihydroxy-4-methylcoumarin	Unknown [32]
	46	12.61	M + HCO_2_	441.1766	441.1754	−2.8004	C_20_H_28_O_8_	89.0241 [M-C_13_H_22_O_8_]^−^ 159.0811 [M-H-C_6_H_10_O_6_-CH_2_O-C_3_H_6_O]^−^ 185.0963 [M-H-C_6_H_10_O_6_-CH_2_O]^−^	Lobetyolin	*Platycodon grandiflorus* (Jacq.) A. DC. [36]
	47	14.86	M + H	209.0808	209.0799	−4.5197	C_11_H_12_O_4_	134.0593 [M-H-C_2_H_4_O-CH_3_]^+^ 149.0492 [M-H-C_2_H_4_O_2_]^+^ 177.0540 [M-H-CH_4_O]^+^	Ferulic acid methyl ester	Unknown [12]

*Note.* ^a^Identification confirmed with reference compound.

## Data Availability

The data used to support the findings of this study are available from the corresponding author upon request.
